# Age at menarche and its relationship to body mass index among adolescent girls in Kuwait

**DOI:** 10.1186/1471-2458-13-29

**Published:** 2013-01-12

**Authors:** Nora Al-Awadhi, Nouf Al-Kandari, Teebah Al-Hasan, Daliah AlMurjan, Salhah Ali, Abdullah Al-Taiar

**Affiliations:** 1 Dept. Community Medicine and Behavioural Sciences, Faculty of Medicine, Kuwait University, Box: 24923, Safat, 13110, Kuwait

**Keywords:** Age at menarche, Kuwait, Gulf, Obesity, Overweight, Girls, Puberty

## Abstract

**Background:**

Despite the increasing rates of childhood obesity and rapid change in socio-economic status, the mean age at menarche remains mostly unknown among contemporary girls in Kuwait and other countries in the Gulf region. This study aimed to estimate the mean age at menarche among schoolgirls in Kuwait and investigate the association between age at menarche and obesity.

**Methods:**

A cross-sectional study was conducted on 1,273 randomly selected female high school students from all governorates in Kuwait. Overweight was defined as higher than or equal to the 85th percentile and obesity as higher than or equal to the 95th percentile using growth charts provided by the Centres for Disease Control and Prevention (CDC, 2000). Data on menarche, socio-demographic status, physical activity and diet were collected using confidential self-administered questionnaire.

**Results:**

Out of 1,273 students, 23 (1.8%) were absent or refused to participate. The mean age at menarche was 12.41 years (95% CI: 12.35-12.48). The prevalence of early menarche, defined as less than 11 years of age, was 8.5% (95% CI: 7.0-10.2%). The prevalence of obesity and overweight was 18.3% (95% CI: 16.2-20.6%) and 25.8% (95% CI: 23.42-28.30%), respectively. Age at menarche was inversely and significantly associated with odds of overweight and obesity after adjusting for potential confounders, odds ratio 0.84 (0.77-0.93); (p = 0.001).

**Conclusion:**

Age at menarche among contemporary girls in Kuwait is similar to that in industrialized countries. There is an inverse association between age at menarche and obesity or overweight. Trends in menarcheal age should be monitored and time of sexual maturation and its related factors should be taken into account in strategies that aim to combat obesity.

## Background

Menarche is the onset of menstruation and is one of the milestones in women’s lives. Although it is a late marker of puberty, it is a well validated indicator and an easily remembered event when compared to other events in the process of female sexual maturity [1,2]. The mean age at menarche varies from one setting to another and is known to be a sensitive indicator of various characteristics of population including socio-economic status, nutritional status, geographical location and environmental conditions. Studying age at menarche is not derived by academic interest, but rather by the huge public health implications which might be caused by changes in the mean age at menarche. Early age at menarche has been linked to several adverse health effects during childhood such as eating disorders [3] and depression [4]; and during adulthood such as type 2 diabetes [5-8], metabolic syndrome [9], breast cancer [10-12], cardiovascular disease and overall mortality [13-15]. Estimating age at menarche is also critical for patient education and may guide the clinical evaluation to identify deviations from normal [16].

Age at menarche appears to have been declining in recent decades in western industrialized countries [2,17-21] because of several plausible factors one of which is the increasing rates of childhood obesity which has been postulated to be the prime factor [22-25]. A similar or even larger increase in the rates of childhood obesity has been noted over recent decades in the oil-rich countries in the Gulf region [26-29] but it is not clear if this was associated with a decline in the age at menarche. Moreover, socio-economic status, which is another determinant of the age at menarche, has changed dramatically in the area because of the increase in the oil revenues in recent years. The data on the mean age at menarche in industrialized countries are abundant but the data from the oil-rich countries in the Gulf region or the broader Middle East are scarce, and the age at menarche among contemporary girls remains practically unknown. We have conducted a cross-sectional study to estimate the age at menarche among girls in Kuwait and investigate the association between age at menarche and obesity or overweight.

## Methods

Kuwait is a small country with a total population of 3.4 million; with approximately 27% of the population under 20 years. There are 124,779 students enrolled in 114 high schools in Kuwait, 56.73% of whom are females. The study population comprises females attending public high schools in all governorates in Kuwait, which typically include females aged between 15 and 19 years. School enrollment for both females and males is extremely high in Kuwait, with an illiteracy rate of only 0.02% among Kuwaiti females aged 15–19 years.

Data on the age at menarche and potential confounders, including physical activity and dietary intake, were collec-ted by self-administered questionnaires. Self-administered questionnaire was used rather than personal interview because cultural norms in the Middle East may prevent the discussion of this topic which is deemed to be sensitive [30]. Particular attention was given to phrasing the questions regarding age at menarche. Three consecutive questions were prepared, including age at menarche, date at which menarche has occurred and, finally, school grade at which menarche has occurred. These three questions were used to validate the reported data on age at menarche. Data on dietary factors were collected including frequency of breakfasts, lunches and dinners not prepared at home per week, and frequency of fast food meals consumed per week; in addition to the daily intake (servings per day) of eggs, pasta or rice, salty snacks or sweets, carbonated beverages, dairy products, meats, fruits, vegetables and salad. Data were collected on physical activities such as the amount of time spent on running/jogging, biking, swimming, or other sports per week over the past year, the number of attended gym classes per week; in addition to the number of hours spent on reading/doing homework during weekdays and weekends, amount of time spent watching TV, using the computer or internet, and playing video games both during weekdays and weekends. The questionnaire was developed in English and then translated to Arabic. The Arabic version of the questionnaire was tested on 20 female students, who subsequently were not included in the study. The aim was to detect ambiguity and estimate the time required to fill the questionnaire. Only a few modifications were made after this process. In order to encourage more candid responses, participants completed the questionnaire in confidence with their peers unable to see their answers and all field workers were female senior medical students. We performed all anthropometric measures either in the schools’ clinics or other private spaces in the schools. Height was measured to the nearest 0.5 cm with a portable stadiometer (SECA^TM^). Weight was measured using a portable digital weight scale (Omron^TM^) to the nearest 100 grams with shoes and heavy clothing removed.

In order to select a representative sample of female students at public high schools in all governorates of Kuwait, a multistage random sampling was used. A list of all public high schools in all governorates in Kuwait was obtained from the Ministry of Education. This was used to select a random sample of schools by using randomly generated numbers. In each governorate, the number of schools was selected according to the number of students required, which was based on the relative size of that governorate, judged by the number of adolescents in the age group (15–19 years). The average number of female students in each high school is approximately 350 students. In order to recruit the required sample (1300 female students), it was decided to recruit 25% of the students in each school, as a result, students were selected from 15 high schools. In each high school, we selected students using a systematic random sample with the list of students as a sampling frame. We used a calculator to generate a random number as a starting point, and then every fourth student was selected.

Data were entered and analyzed using Statistical Package for Social Sciences (SPSS) version 17. Body Mass Index (BMI) was calculated as weight (kg) ÷ Height^2^ (m). BMI-for-age was calculated by means of weight, height, age, and gender, as determined by the growth charts provided by the Centers for Disease Control and Prevention (CDC, 2010) using inbuilt function in STATA StataCorp. Overweight and obesity were defined as more than or equal to 85th BMI and 95th BMI percentile, respectively, while underweight was defined as less than or equal to 5th BMI percentile. The term overweight in this study does not include obese girls. We calculated age at menarche by subtracting the date of birth from the date at which menarche has occurred; 533 (42.6%) participants were able to report the exact date. Those who were unable to report the exact date at which menarche has occurred reported their age at menarche and/or their school grade. Only 24 participants could not remember their age at menarche precisely and reported instead the school grade at which menarche was attained. We tested the differences in means by using Analysis of Variance (ANOVA) or Kruskal-Wallis as appropriate. The 95% confidence intervals for the mean age at menarche and the prevalence of obesity or overweight were calculated by normal approximation and binomial distribution, respectively.

Unconditional binary logistic regression was used to investigate the association between BMI categories (obesity/overweight vs. normal or underweight) and age at menarche divided into tertiles while adjusting for various confounders. Confounding variables were categorized into three groups, namely, socio-demographic factors, physical activity and dietary intake factors. These groups of confounders were adjusted for, and their impact on the crude odds ratio was noted. Only factors showing significant association with the outcome in the univariate analysis were considered to be potential confounders. Statistical significance of the association between age at menarche and obesity or overweight was tested using the likelihood ratio test, comparing the model with and without the variable. The analysis was also repeated with age at menarche fitted as a continuous variable and the findings were reported in the text. Written informed consent was obtained from the participants and the approval of the Ministry of Education and the school principals were obtained. The study was ethically approved at the Department of Community Medicine and Behavioral Sciences, Kuwait University.

## Results

By the end of the study, 1,273 female students were selected, of whom 23 (1.8%) were absent or refused to participate. (Table 1) shows the socio-demographic characteristics of the study group. The mean (SD) age of the study group was 16.4 (1.15) years with the majority of students being Kuwaiti 1,175 (94.0%). Most of the participants 1,054 (84.3%) live with both parents and more than one-third of the students came from families with parental education equal to university or above. 

**Table 1:** Socio-demographic characteristics of 1,250 female high-school students in Kuwait, 2010

**Characteristics**		
Age in years, Mean (SD)	16.4 (1.15)	
	**n**	**(%)**
Nationality		
Kuwaiti	1175	(94.0)
Non-Kuwaiti	75	(6.0)
Father’s Education^1^		
No formal education	35	(2.8)
Primary/intermediate	210	(16.8)
Secondary	392	(31.4)
Diploma	163	(13.0)
University and above	436	(34.9)
Mother’s Education^2^		
No formal education	91	(7.3)
Primary/intermediate	185	(14.8)
Secondary	380	(30.4)
Diploma	184	(14.7)
University and above	403	(32.2)
Current father Income (Kuwaiti Dinars)		
≤1000	327	(26.2)
1001-1500	187	(15.0)
≥ 1501	158	(12.6)
Don’t know	578	(46.2)
Current Residence		
With both parents	1054	(84.3)
With mother alone	155	(12.4)
With father alone	22	(1.8)
Other relatives only	19	(1.5)

^1^ Missing for 14 participants, ^2^ Missing for 7 participants.

The mean (SD) age at menarche was 12.41 (1.24) years (95% CI: 12.35-12.48). There was no significant difference in the mean age at menarche between Kuwaitis and non-Kuwaitis, 12.41 (1.23) years and 12.48 (1.42) years, respectively (p = 0.62). Early menarche, defined as less than 11 years of age [31], was found in 106 (8.5%) of the participants (95% CI: 7.0%-10.2%). Six female students had not attained menarche by the time of the study one of whom has a diagnosis of systemic lupus erythematosus. Their median age was 15.6 (Interquartile Range; IQR: 14.8-15.9) years. There were 12 female students who attained menarche but reported diagnosed conditions of diabetes (5 girls), congenital heart disease (1 girl) and thyroid problems (2 girls).

**Figure 1:**
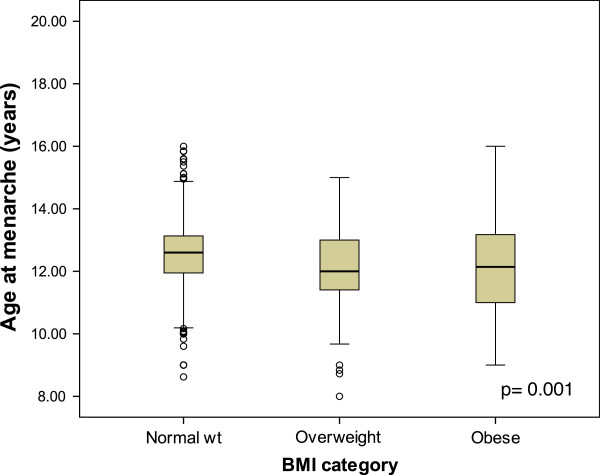
Age at menarche in different BMI categories of 1,250 female high school students in Kuwait, 2010.

The prevalence of obesity among female high school students was 18.3% (95% CI: 16.2%-20.6%), while the prevalence of overweight was 25.8% (95% CI: 23.4%-28.3%). (Figure 1) shows the median age at menarche among obese, overweight and normal-weight students. Median age at menarche was significantly lower among students who were overweight, 12.00 years (IQR: 11.41-13.00) or obese, 12.19 years (IQR: 11.00-13.17) compared to those with normal BMI, 12.61 years (IQR: 11.95-13.14) (p = 0.001). Only 12 (0.96%) female students were underweight and their median age at menarche was 13.00 (IQR: 12.00-14.06) years. The prevalence of obesity or overweight were 51.1%, 43.0% and 40.0% among those who had their menarche in the age <12 years, 12 to less than 13 years, and 13 years or above, respectively (Chi-square for trend, p = 0.001). 

(Table 2) shows the association between obesity or overweight (obesity/overweight vs. normal/underweight) and age at menarche before and after adjusting for potential confounders. Confounders were categorized into three groups that included socio-demographic characteristics, factors related to physical activity and dietary factors. In order to adjust for confounding by these variables, these groups were added to the model sequentially. The association between age at menarche and obesity or overweight remained evident and significant after adjusting for all potential confounders. The odds of being obese or overweight among girls with age at menarche of 13 years or above was 0.65 times (95% CI: 0.49-0.87) the odds of those with age at menarche less than 12 years (p = 0.012). We repeated the analysis in Table 2 with age at menarche fitted as a continuous variable. Age at menarche was inversely and significantly associated with odds of obesity or overweight before and after adjusting for potential confounders, adjusted odds ratio 0.84 (0.77-0.93); (p = 0.001). 

**Table 2:** Association between obesity or overweight (obesity/overweight vs. normal/underweight) and age at menarche, using multivariate logistic regression

	**Odds ratio (95%CI)**			
**Age at menarche**	**Model 1**	**Model 2**	**Model 3**	**Model 4**
Less than 12 years	Ref.	Ref.	Ref.	Ref.
12 to less than 13 years	0.72 (0.54-0.96)	0.71 (0.53-0.96)	0.73 (0.54-0.99)	0.71 (0.52-0.97)
13 years or above	0.64 (0.48-0.84)	0.64 (0.49-0.85)	0.67 (0.50-0.88)	0.65 (0.49-0.87)
**P-value**	0.005	0.006	<0.001	0.012

Model 1: unadjusted. Model 2 adjusted for Group 1 (i.e. area of residence, living with both parents vs. single parent or a relative). Model 3: adjusted for Group 1 and Group 2 (i.e. presence of disease condition that limits physical activity, amount of running/jogging over the past year, number of hours spent reading/doing homework during weekdays). Model 4: adjusted for Group 1, Group 2 and Group 3 (frequency of dinners prepared away from home, frequency of fast-food intake, frequency of eggs intake, number of servings per day (divided into tertiles) of pasta and rice, number of servings per day of salty snacks and sweets).

## Discussion

Only few studies have investigated the age at menarche in oil-rich countries in the Gulf region [30-33]. These studies are old and were conducted among adult women whose reported age at menarche is less reliable [34]. Thus, the age at menarche among contemporary girls remained unknown, despite the dramatic changes in socio-economic status and the increasing rates of childhood obesity in the region. Principles of prevention, including Rose Theorem, suggest that small changes in the mean age at menarche will have an enormous impact on public health. This study aimed to estimate the mean age at menarche among contemporary girls in Kuwait and investigate the association between age at menarche and obesity or overweight.

Age at menarche among contemporary girls was estimated to be 12.41 years (95% CI: 12.35-12.48), which is slightly lower than that reported more than a decade ago. While studying the important causes of anaemia among girls in secondary schools in Kuwait, Jackson and Al-Mousa [33] estimated the age at menarche to be 12.7 years. It is not clear whether the difference represents a genuine decline in age at menarche similar to that reported in the US [35] (from 12.53 years in 1988/1994 to 12.34 years in 1999/2002), Japan [36] (from 12.3 years for women born 1960 to 12.2 years for women born in 1980), Croatia [37] (from 12.92 years in 1997 to 12.31 years in 2010) and Ireland [38] (from 13.52 years in 1986 to 12.53 years in 2006) or just reflects different methodological approaches. Although there is no data that allow for direct comparison from the region, more than two decades ago, in a cancer survey in Saudi Arabia, a difference of 0.4 year in the age at menarche was reported between women born 20 years ago and women born 45 years ago [32]. Recent reports in western countries suggest that the age at menarche is no longer declining and that it has started to level off [39,40]. With the rapid change in socio-economic status in the region and increasing rates of childhood obesity, it is critical to monitor the trends in the age at menarche by longitudinal studies. Barriers to studying age at menarche that have been described before [30] seem to have disappeared among contemporary girls in the region.

The current estimate of age at menarche in Kuwait seems to be similar to the current estimates of age at menarche among high school students in most western countries such as Italy (12.4 years) [39], US (12.3 years for black girls and 12.6 years for white girls) [41], and UK (12.5 years) [42]; but seems to be lower than that reported from most low-income countries such as Nigeria (15.26 years) [43], India (13.22 years) [44], Bangladesh (13.12 years) [45], Ghana (12.74 years) [46], Ethiopia (16.9 years) [47], Turkey (13.04 years) [48], Argentina (12.84 years) [49], Indonesia (12.96 years) [50], and Tanzania (14.8 years) [51]. Nevertheless, our estimate is similar to that reported among Egyptian girls in secondary schools (12.44 years) [52]. Although comparing age at menarche between different settings has attracted attention in recent years, variation of sexual maturation by race or ethnicity is well documented [41,53], thus comparing menarcheal age across racially diverse groups would probably be inappropriate.

The prevalence of early menarche, defined as less than 11 years of age, was 8.6%. This is similar to that reported for White American girls (7.8%) [54] but lower than that reported for Black (12.3%) and Hispanic (13.6%) American girls [54]. However, the prevalence of early menarche depends on the age limit that is used to define early menarche which is neither definitive nor universal. Some authors have suggested using 5th percentile as an indication for anomalies in pubertal timing [55], which is 10.4 years in our study. Other studies have defined early menarche as one standard deviation below their estimated mean of age at menarche [56]; according to this definition approximately 1 out of 5 girls in our study would have an early menarche.

One of the primary objectives of this study was to examine the association between age at menarche and obesity or overweight among contemporary girls in Kuwait. There was a significant inverse association between age at menarche and obesity or overweight before and after adjusting for potential confounders, (Table 2). This is consistent with recent findings that showed an inverse association between age at menarche and BMI among school girls, which was evident across all socio-economic groups [39,57,58]. Our findings are also consistent with that reported from the United States [24,59], Croatia [60], and Chile [61] where early sexual maturation was associated with obesity among school girls. There are several explanations for the association between age at menarche and obesity, but the specific mechanisms remain unclear. One of the possible explanations is that higher levels of prepubertal BMI lead to an increase in the production and availability of estrogen through various mechanisms, which predisposes to early menarche [62]. Another explanation suggests that early menarche associates with higher levels of estrogen which increase fat deposition in peripheral adipose tissues [55]. It is also possible that genetic predisposition for early menarche and obesity are both influenced by independent genetic factors [63] but, to date, no specific gene has been identified to be responsible for age at menarche in all ethnicities [64].

School enrolment is extremely high in Kuwait which suggests that it is possible to generalize our findings to all girls in public schools in Kuwait, but our findings cannot be extrapolated to adolescents in private schools. It is possible that girls in private schools have even earlier age at menarche because of the high socio-economic status of this particular group although socio-economic factors do not seem to play as significant a role as in the past [39]. We have used confidential self-administered questionnaire rather than personal interview which resulted in a higher response rate (only 1.8% were absent or refused to participate). Although studies assessing the accuracy of the reported age at menarche by adult women have shown different results [34,65-67], age at menarche reported by schoolgirls is more reliable. In our study, more than 42% of the participants were able to remember the exact date at which menarche had occurred, and the estimated age at menarche from this subgroup was identical to that estimated from the whole study group. We have also explored the impact of the six girls who had not attained menarche by the time of the study by assuming their age at menarche to be the maximum age at menarche in the study group. We also explored exclusion of the girls with disease conditions that may interfere with menarche. Finally, we have collected data on physical activity and dietary intake using locally developed questions which may result in some residual confounding in studying the relationship between age at menarche and obesity.

## Conclusion

In conclusion, the mean age at menarche among contemporary girls in Kuwait was 12.41 years. There is no previous study with adequate methodology to compare and ascertain whether age at menarche has declined over the last few decades in Kuwait or other countries in the Gulf region. There was a significant inverse relationship between age at menarche and obesity or overweight before and after adjusting for potential confounders. Although these findings are consistent with that reported from other regions, they have particular importance in our setting, because of the extremely high rates of childhood obesity and Type 2 diabetes. Changes in age at menarche have important health implications which highlight the need to monitor the trends in age at menarche in Kuwait and other countries in the Gulf region, where lifestyle, socio-economic and dietary factors are rapidly changing. Strategies that combat obesity among adolescents and young adults should take into account early sexual maturation and its consequences as well as environmental factors that may predispose to early menarche.

## Competing interests

The authors declare that they have no competing interests.

## Authors’ contributions

NAA: Contributed to the study design, data collection, data analysis and data interpretation. NAK: Contributed to the study design, data collection, data analysis and data interpretation. TAH: Contributed to data collection, data analysis and revised the manuscript. DAM: Contributed to data collection, data analysis and revised the manuscript. SEA: Contributed to data collection, data analysis and revised the manuscript. AAT: Design the study, supervised data collection and data analysis, drafted the paper. All authors read and approved the final manuscript.

## Pre-publication history

The pre-publication history for this paper can be accessed here:

http://www.biomedcentral.com/1471-2458/13/29/prepub

## References

[B1] Herman-Giddens ME Slora EJ Wasserman RC Bourdony CJ Bhapkar MV Koch GG Hasemeier CM Secondary sexual characteristics and menses in young girls seen in office practice: a study from the pediatric research in office settings network Pediatrics 1997 99 4 505 512 9093289 10.1542/peds.99.4.505

[B2] Parent AS Teilmann G Juul A Skakkebaek NE Toppari J Bourguignon JP The timing of normal puberty and the age limits of sexual precocity: variations around the world, secular trends, and changes after migration Endocr Rev 2003 24 5 668 693 14570750 10.1210/er.2002-0019

[B3] Striegel-Moore RH McMahon RP Biro FM Schreiber G Crawford PB Voorhees C Exploring the relationship between timing of menarche and eating disorder symptoms in Black and White adolescent girls Int J Eat Disord 2001 30 4 421 433 11746303 10.1002/eat.1103

[B4] Kaltiala-Heino R Kosunen E Rimpela M Pubertal timing, sexual behaviour and self-reported depression in middle adolescence J Adolesc 2003 26 5 531 545 12972267 10.1016/s0140-1971(03)00053-8

[B5] Dreyfus JG Lutsey PL Huxley R Pankow JS Selvin E Fernandez-Rhodes L Franceschini N Demerath EW Age at menarche and risk of type 2 diabetes among African-American and white women in the Atherosclerosis Risk in Communities (ARIC) study Diabetologia 2012 55 9 2371 2380 22760786 10.1007/s00125-012-2616-z PMC3690318

[B6] Stockl D Doring A Peters A Thorand B Heier M Huth C Stockl H Rathmann W Kowall B Meisinger C Age at menarche is associated with prediabetes and diabetes in women (aged 32–81 years) from the general population: the KORA F4 Study Diabetologia 2012 55 3 681 688 22170465 10.1007/s00125-011-2410-3

[B7] He C Zhang C Hunter DJ Hankinson SE Buck Louis GM Hediger ML Hu FB Age at menarche and risk of type 2 diabetes: results from 2 large prospective cohort studies Am J Epidemiol 2010 171 3 334 344 20026580 10.1093/aje/kwp372 PMC2842205

[B8] Lakshman R Forouhi N Luben R Bingham S Khaw K Wareham N Ong KK Association between age at menarche and risk of diabetes in adults: results from the EPIC-Norfolk cohort study Diabetologia 2008 51 5 781 786 18320165 10.1007/s00125-008-0948-5

[B9] Stockl D Meisinger C Peters A Thorand B Huth C Heier M Rathmann W Kowall B Stockl H Doring A Age at menarche and its association with the metabolic syndrome and its components: results from the KORA F4 study PLoS One 2011 6 10 e26076 22028807 10.1371/journal.pone.0026076 PMC3196515

[B10] Trentham-Dietz A Nichols HB Remington PL Yanke L Hampton JM Newcomb PA Love RR Correlates of age at menarche among sixth grade students in Wisconsin WMJ: official publication of the State Medical Society of Wisconsin 2005 104 7 65 69 16294603

[B11] Petridou E Syrigou E Toupadaki N Zavitsanos X Willett W Trichopoulos D Determinants of age at menarche as early life predictors of breast cancer risk International journal of cancer Journal international du cancer 1996 68 2 193 198 8900427 10.1002/(SICI)1097-0215(19961009)68:2<193::AID-IJC9>3.0.CO;2-T

[B12] Hadjisavvas A Loizidou MA Middleton N Michael T Papachristoforou R Kakouri E Daniel M Papadopoulos P Malas S Marcou Y An investigation of breast cancer risk factors in Cyprus: a case control study BMC Cancer 2010 10 447 20727220 10.1186/1471-2407-10-447 PMC2933629

[B13] Jacobsen BK Heuch I Kvale G Association of low age at menarche with increased all-cause mortality: a 37-year follow-up of 61,319 Norwegian women Am J Epidemiol 2007 166 12 1431 1437 17875585 10.1093/aje/kwm237

[B14] Lakshman R Forouhi NG Sharp SJ Luben R Bingham SA Khaw KT Wareham NJ Ong KK Early age at menarche associated with cardiovascular disease and mortality J Clin Endocrinol Metab 2009 94 12 4953 4960 19880785 10.1210/jc.2009-1789

[B15] Jacobsen BK Oda K Knutsen SF Fraser GE Age at menarche, total mortality and mortality from ischaemic heart disease and stroke: the Adventist Health Study, 1976–88 Int J Epidemiol 2009 38 1 245 252 19188208 10.1093/ije/dyn251 PMC2722816

[B16] Diaz A Laufer MR Breech LL Menstruation in girls and adolescents: using the menstrual cycle as a vital sign Pediatrics 2006 118 5 2245 2250 17079600 10.1542/peds.2006-2481

[B17] de Muinich Keizer SM Mul D Trends in pubertal development in Europe Hum Reprod Update 2001 7 3 287 291 11392375 10.1093/humupd/7.3.287

[B18] Whincup PH Gilg JA Odoki K Taylor SJ Cook DG Age of menarche in contemporary British teenagers: survey of girls born between 1982 and 1986 BMJ 2001 322 7294 1095 1096 11337438 10.1136/bmj.322.7294.1095 PMC31261

[B19] Herman-Giddens ME The decline in the age of menarche in the United States: should we be concerned? The Journal of adolescent health: official publication of the Society for Adolescent Medicine 2007 40 3 201 203 17321418 10.1016/j.jadohealth.2006.12.019

[B20] Lehmann A Scheffler C Hermanussen M The variation in age at menarche: an indicator of historic developmental tempo Anthropologischer Anzeiger; Bericht uber die biologisch-anthropologische Literatur 2010 68 1 85 99 10.1127/0003-5548/2010/0086 20954458

[B21] Cabanes A Ascunce N Vidal E Ederra M Barcos A Erdozain N Lope V Pollan M Decline in age at menarche among Spanish women born from 1925 to 1962 BMC Publ Health 2009 9 449 10.1186/1471-2458-9-449 PMC2796666 19961593

[B22] Currie C Ahluwalia N Godeau E Nic Gabhainn S Due P Currie DB Is obesity at individual and national level associated with lower age at menarche? evidence from 34 countries in the health behaviour in school-aged children study The Journal of adolescent health: official publication of the Society for Adolescent Medicine 2012 50 6 621 626 22626490 10.1016/j.jadohealth.2011.10.254

[B23] Ahmed ML Ong KK Dunger DB Childhood obesity and the timing of puberty Trends in endocrinology and metabolism: TEM 2009 20 5 237 242 19541497 10.1016/j.tem.2009.02.004

[B24] Kaplowitz PB Slora EJ Wasserman RC Pedlow SE Herman-Giddens ME Earlier onset of puberty in girls: relation to increased body mass index and race Pediatrics 2001 108 2 347 353 11483799 10.1542/peds.108.2.347

[B25] Walvoord EC The timing of puberty: is it changing? Does it matter? The Journal of adolescent health: official publication of the Society for Adolescent Medicine 2010 47 5 433 439 20970077 10.1016/j.jadohealth.2010.05.018

[B26] Musaiger AO Overweight and obesity in eastern mediterranean region: prevalence and possible causes Journal of obesity 2011 2011 407237 21941635 10.1155/2011/407237 PMC3175401

[B27] Ng SW Zaghloul S Ali HI Harrison G Popkin BM The prevalence and trends of overweight, obesity and nutrition-related non-communicable diseases in the Arabian Gulf States Obesity reviews: an official journal of the International Association for the Study of Obesity 2011 12 1 1 13 20546144 10.1111/j.1467-789X.2010.00750.x

[B28] Guy W Nunn V Thomas E Bell J Obesity, diabetes and longevity in the Gulf: Is there a Gulf Metabolic Syndrome? International Journal of Diabetes Mellitus 2009 1 1 43 54

[B29] Abdelalim A Ajaj N Al-Tmimy A Alyousefi M Al-Rashaidan S Hammoud MS Al-Taiar A Childhood obesity and academic achievement among male students in public primary schools in Kuwait Medical principles and practice: international journal of the Kuwait University, Health Science Centre 2012 21 1 14 19 10.1159/000331792 22024698

[B30] Badrinath P Ghazal-Aswad S Parfitt D Osman N Cultural and ethnic barriers in conducting research. Factors influencing menarche in the United Arab Emirates Saudi medical journal 2004 25 11 1626 1630 15573190

[B31] Babay ZA Addar MH Shahid K Meriki N Age at menarche and the reproductive performance of Saudi women Ann Saudi Med 2004 24 5 354 356 15573847 10.5144/0256-4947.2004.354 PMC6148136

[B32] Jabbar FA Wong SS Age at menarche and reproductive pattern among Saudi women J R Soc Health 1988 108 3 94 96 3135403 10.1177/146642408810800308

[B33] Jackson RT Al-Mousa Z Iron deficiency is a more important cause of anemia than hemoglobinopathies in Kuwaiti adolescent girls J Nutr 2000 130 5 1212 1216 10801921 10.1093/jn/130.5.1212

[B34] Cooper R Blell M Hardy R Black S Pollard TM Wadsworth ME Pearce MS Kuh D Validity of age at menarche self-reported in adulthood J Epidemiol Community Health 2006 60 11 993 997 17053289 10.1136/jech.2005.043182 PMC2465480

[B35] Anderson SE Must A Interpreting the continued decline in the average age at menarche: results from two nationally representative surveys of U.S. girls studied 10 years apart J Pediatr 2005 147 6 753 760 16356426 10.1016/j.jpeds.2005.07.016

[B36] Hosokawa M Imazeki S Mizunuma H Kubota T Hayashi K Secular trends in age at menarche and time to establish regular menstrual cycling in Japanese women born between 1930 and 1985 BMC Womens Health 2012 12 1 19 22800445 10.1186/1472-6874-12-19 PMC3434095

[B37] Vecek N Vecek A Zajc Petranovic M Tomas Z Arch-Vecek B Skaric-Juric T Milicic J Secular trend of menarche in Zagreb (Croatia) adolescents Eur J Obstet Gynecol Reprod Biol 2012 160 1 51 54 22000342 10.1016/j.ejogrb.2011.09.029

[B38] O’Connell A Gavin A Kelly C Molcho M Nic Gabhainn S The mean age at menarche of Irish girls in 2006 Ir Med J 2009 102 3 76 79 19489194

[B39] Rigon F Bianchin L Bernasconi S Bona G Bozzola M Buzi F Cicognani A De Sanctis C De Sanctis V Radetti G Update on age at menarche in Italy: toward the leveling off of the secular trend The Journal of adolescent health: official publication of the Society for Adolescent Medicine 2010 46 3 238 244 20159500 10.1016/j.jadohealth.2009.07.009

[B40] Papadimitriou A Fytanidis G Douros K Bakoula C Nicolaidou P Fretzayas A Age at menarche in contemporary Greek girls: evidence for levelling-off of the secular trend Acta paediatrica (Oslo, Norway: 1992) 2008 97 6 812 815 10.1111/j.1651-2227.2008.00806.x 18460111

[B41] Freedman DS Khan LK Serdula MK Dietz WH Srinivasan SR Berenson GS Relation of age at menarche to race, time period, and anthropometric dimensions: the Bogalusa Heart Study Pediatrics 2002 110 4 e43 12359816 10.1542/peds.110.4.e43

[B42] Joinson C Heron J Lewis G Croudace T Araya R Timing of menarche and depressive symptoms in adolescent girls from a UK cohort The British journal of psychiatry: the journal of mental science 2011 198 1 17 23 sup 11–12 21200072 10.1192/bjp.bp.110.080861

[B43] Tunau KA Adamu AN Hassan MA Ahmed Y Ekele BA Age at menarche among school girls in Sokoto, Northern Nigeria Ann Afr Med 2012 11 2 103 107 22406670 10.4103/1596-3519.93533

[B44] Deb R Age at menarche in adolescent Khasi girls, Meghalaya Indian Pediatr 2011 48 1 69 21317473

[B45] Hossain MG Islam S Aik S Zaman TK Lestrel PE Age at menarche of university students in Bangladesh: secular trends and association with adult anthropometric measures and socio-demographic factors J Biosoc Sci 2010 42 5 677 687 20529410 10.1017/S0021932010000210

[B46] Aryeetey R Ashinyo A Adjuik M Age of menarche among basic level school girls in Medina, Accra Afr J Reprod Health 2011 15 3 103 110 22574497

[B47] Zegeye DT Megabiaw B Mulu A Age at menarche and the menstrual pattern of secondary school adolescents in northwest Ethiopia BMC Womens Health 2009 9 29 19804623 10.1186/1472-6874-9-29 PMC2763859

[B48] Ekerbicer HC Celik M Kiran H Kiran G Age at menarche in Turkish adolescents in Kahramanmaras, Eastern Mediterranean region of Turkey The European journal of contraception & reproductive health care: the official journal of the European Society of Contraception 2007 12 3 289 293 10.1080/13625180701447854 17763268

[B49] Orden AB Vericat A Apezteguia MC Age at menarche in urban Argentinian girls: association with biological and socioeconomic factors Anthropologischer Anzeiger; Bericht uber die biologisch-anthropologische Literatur 2011 68 3 309 322 10.1127/0003-5548/2011/0109 21905419

[B50] Batubara JR Soesanti F van de Waal HD Age at menarche in indonesian girls: a national survey Acta Med Indones 2010 42 2 78 81 20513931

[B51] Rebacz E Age at menarche in schoolgirls from Tanzania in light of socioeconomic and sociodemographic conditioning Coll Antropol 2009 33 1 23 29 19408599

[B52] Ghaly I Hussein FH Abdelghaffar S Anwar G Seirvogel RM Optimal age of sexual maturation in Egyptian children Eastern Mediterranean health journal = La revue de sante de la Mediterranee orientale = al-Majallah al-sihhiyah li-sharq al-mutawassit 2008 14 6 1391 1399 19161115

[B53] Reagan PB Salsberry PJ Fang MZ Gardner WP Pajer K African-American/white differences in the age of menarche: Accounting for the difference Soc Sci Med 2012 75 7 1263 1270 22726619 10.1016/j.socscimed.2012.05.018 PMC3407312

[B54] Adair LS Gordon-Larsen P Maturational timing and overweight prevalence in US adolescent girls Am J Public Health 2001 91 4 642 644 11291382 10.2105/ajph.91.4.642 PMC1446647

[B55] Gaudineau A Ehlinger V Vayssiere C Jouret B Arnaud C Godeau E Factors associated with early menarche: results from the French Health Behaviour in School-aged Children (HBSC) study BMC Publ Health 2010 10 175 10.1186/1471-2458-10-175 PMC2853511 20353570

[B56] Al-Sahab B Ardern CI Hamadeh MJ Tamim H Age at menarche in Canada: results from the National Longitudinal Survey of Children & Youth BMC Publ Health 2010 10 736 10.1186/1471-2458-10-736 PMC3001737 21110899

[B57] Wronka I Association between BMI and age at menarche in girls from different socio-economic groups Anthropologischer Anzeiger; Bericht uber die biologisch-anthropologische Literatur 2010 68 1 43 52 10.1127/0003-5548/2010/0066 20954455

[B58] Hernandez MI Unanue N Gaete X Cassorla F Codner E [Age of menarche and its relationship with body mass index and socioeconomic status] Rev Med Chil 2007 135 11 1429 1436 18259654

[B59] Wang Y Is obesity associated with early sexual maturation? A comparison of the association in American boys versus girls Pediatrics 2002 110 5 903 910 12415028 10.1542/peds.110.5.903

[B60] Bralic I Tahirovic H Matanic D Vrdoljak O Stojanovic-Spehar S Kovacic V Blazekovic-Milakovic S Association of early menarche age and overweight/obesity Journal of pediatric endocrinology & metabolism: JPEM 2012 25 1–2 57 62 10.1515/jpem-2011-0277 22570951

[B61] Amigo H Vasquez S Bustos P Ortiz G Lara M Socioeconomic status and age at menarche in indigenous and non-indigenous Chilean adolescents Cadernos de saude publica / Ministerio da Saude, Fundacao Oswaldo Cruz, Escola Nacional de Saude Publica 2012 28 5 977 983 10.1590/s0102-311x2012000500016 22641520

[B62] Cheng G Buyken AE Shi L Karaolis-Danckert N Kroke A Wudy SA Degen GH Remer T Beyond overweight: nutrition as an important lifestyle factor influencing timing of puberty Nutr Rev 2012 70 3 133 152 22364156 10.1111/j.1753-4887.2011.00461.x

[B63] Mamun AA Hayatbakhsh MR O’Callaghan M Williams G Najman J Early overweight and pubertal maturation–pathways of association with young adults’ overweight: a longitudinal study Int J Obes (Lond) 2009 33 1 14 20 18982007 10.1038/ijo.2008.220

[B64] Dvornyk V Waqar ul H Genetics of age at menarche: a systematic review Hum Reprod Update 2012 18 2 198 210 22258758 10.1093/humupd/dmr050

[B65] Koprowski C Coates RJ Bernstein L Ability of young women to recall past body size and age at menarche Obes Res 2001 9 8 478 485 11500528 10.1038/oby.2001.62

[B66] Koo MM Rohan TE Accuracy of short-term recall of age at menarche Ann Hum Biol 1997 24 1 61 64 9022907 10.1080/03014469700004782

[B67] Must A Phillips SM Naumova EN Blum M Harris S Dawson-Hughes B Rand WM Recall of early menstrual history and menarcheal body size: after 30 years, how well do women remember? Am J Epidemiol 2002 155 7 672 679 11914195 10.1093/aje/155.7.672

